# Supplementary material for: Identification of photoperiod-induced specific miRNAs in the adrenal glands of Sunite sheep (Ovis aries)
Source: Front Vet Sci. 2022 Jul 22;9:888207. doi: 10.3389/fvets.2022.888207 (PMC9354845; doi:10.3389/fvets.2022.888207)
Supplement: Supplementary file 1 [file Data_Sheet_1.ZIP › Supplementary file/miRDeep2 file.zip!/miRDeep2 file/Novel_Structure/SPLP42A2/Novel_472.pdf]

Provisional ID	:	NC_019484.2_42705
Score total	:	1.8
Score for star read(s)	:	-1.3
Score for read counts	:	-0.3
Score for mfe	:	1.8
Score for randfold	:	1.6
Score for cons. seed	:	
Total read count	:	11
Mature read count	:	11
Loop read count	:	0
Star read count	:	0

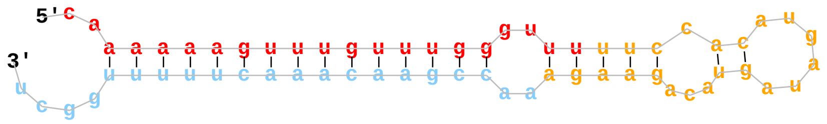

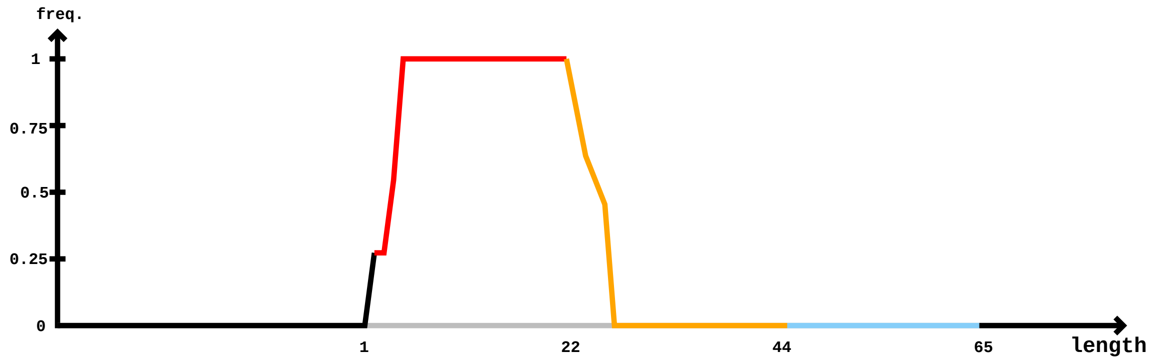

Mature Star

```
5'- agggugaggaacucgguacuauuggggguuggcaaaaaaguuuguuuggguuuuuccacaugauaguacagaagaaaccgaacaaacuuuuuggcuaauccaauagcagc   -3'    exp
    .((((.....))))....((((((((.(((((.(((((((((((((((..(((((.((......))...)))))..))))))))))))))).))))))))))))).....    reads      mm         sample
    ................................caaaaaaguuuguuuggguuu.........................................................    3          0          seq
    ..................................aaaaaguuuguuuggguuuuucc.....................................................    1          0          seq
    ..................................aaaaaguuuguuuggguuuuucU.....................................................    2          1          seq
    ...................................aaaaguuugCuuggguuuu.........................................................    1          1          seq
    ...................................aaaaguuuguuuggguuuuu.......................................................    1          0          seq
    ...................................aaaaguuuguuuggguuuuuc......................................................    1          0          seq
    ...................................Gaaaguuuguuuggguuuuucc.....................................................    2          1          seq
```
